# Effects of transcutaneous electrical nerve stimulation (TENS) on arterial stiffness and blood pressure in resistant hypertensive individuals: study protocol for a randomized controlled trial

**DOI:** 10.1186/s13063-016-1302-8

**Published:** 2016-03-29

**Authors:** José Fernando Vilela-Martin, Luiz Tadeu Giollo-Junior, Gaspar Rogério Chiappa, Gerson Cipriano-Junior, Paulo José Cardoso Vieira, Fábio dos Santos Ricardi, Manoel Ildefonso Paz-Landim, Days Oliveira de Andrade, Elizabeth do Espírito Santo Cestário, Luciana Neves Cosenso-Martin, Juan Carlos Yugar-Toledo, José Paulo Cipullo

**Affiliations:** Internal Medicine Department and Hospital de Base, Hypertension Clinic, Medical School in São José do Rio Preto (FAMERP), Av Anísio Haddad 7700 casa 129, Jd das Palmeiras, 15093-000 São José do Rio Preto, SP Brazil; Cardiology Division, Federal University of Rio Grande do Sul (UFRS), Porto Alegre, Brazil; Physical Therapy Division, University of Brasilia (UNB), Brasilia, Brazil; Cardiology Division, Hospital de Clínicas de Porto Alegre, Porto Alegre, Brazil

**Keywords:** Arterial stiffness, Resistant hypertension, Sympathetic system, Blood pressure, Transcutaneous electrical nerve stimulation

## Abstract

**Background:**

Resistant hypertension (RH) treatment requires an adequate and intense therapeutic approach. However, the results are not always satisfactory despite intensive treatment. Of the different pathophysiological mechanisms involved in the pathogenesis of RH, sympathetic overstimulation and therapies that block the sympathetic system have been widely studied. These approaches, however, are invasive and expensive. Another possible approach is by transcutaneous electrical nerve stimulation (TENS), a noninvasive method that modulates activity by using low-frequency transcutaneous electrical stimulation to inhibit primary afferent pathways. Thus, the current study will evaluate the effect of applying TENS in the cervicothoracic region of subjects with RH and will seek to develop a new low-cost and readily available therapy to treat this group of hypertensive individuals.

**Methods/design:**

This is a randomized, single blind (subject), parallel-assignment study controlled with a sham group and including participants aged 40 to 70 years with resistant hypertension. The trial has two arms: the treatment and control (sham group). The treatment group will be submitted to the stimulation procedure (TENS). The sham group will not be submitted to stimulation. The primary outcomes will be a reduction in the peripheral blood pressure and adverse events. The secondary outcomes will be a reduction the central blood pressure. The study will last 30 days. The sample size was calculated assuming an alpha error of 5 % to reject the null hypothesis with a statistical power of 80 %, thereby resulting in 28 participants per group (intervention versus sham).

**Discussion:**

In recent decades, RH has become very common and costly. Adequate control requires several drugs, and in many cases, treatment is not successful. Sympathetic nervous system inhibition by renal denervation and central inhibition have significant effects in reducing BP; however, these treatments are costly and invasive. Another type of sympathetic nervous system inhibition can also be noninvasively achieved by electric current. Therefore, the application of TENS may be a new therapeutic option for treating resistant hypertensive individuals.

**Trial Registration:**

Clinical Trials NCT02365974

**Electronic supplementary material:**

The online version of this article (doi:10.1186/s13063-016-1302-8) contains supplementary material, which is available to authorized users.

## Background

### Hypertension in the context of cardiovascular disease

In Brazil, cardiovascular diseases (CVD) accounted for 30 % of deaths in 2008, with hypertension alone affecting more than 36 million people [1]. In the United States, this figure is even higher, as approximately 80 million Americans have hypertension [2].

As one of the main risk factors for the development of CVD, hypertension is the starting point of cardiovascular, cerebrovascular, and renal impairment. In most cases, by the time there is a manifestation, this disease, although still manageable, is irreversible. The main risk factors associated with hypertension are smoking, physical inactivity, stress, alcohol consumption, age, obesity, and an unhealthy diet [3], all of which can alter the integrity of the vascular endothelium and lead to a number of complications.

Currently, a broad therapeutic arsenal is available for the treatment of hypertension. Nonetheless, sometimes hypertension becomes difficult to control due to varying situations, thereby characterizing resistant hypertension (RH), which requires a more intensive therapeutic approach. RH is a condition in which the blood pressure (BP) remains above the recommended target even when treated with the maximum tolerated doses of three antihypertensive drugs with synergistic actions, including a diuretic. A condition exists that is known as controlled resistant hypertension, which is defined as high BP that is controlled using four antihypertensive drugs [4]. Although the exact prevalence of RH has not yet been precisely established, this state is estimated to occur in 12–15 % of the hypertensive population [5]. The risk factors associated with RH are similar to those of hypertension; however, it is more common in elderly patients; females; Blacks; obese patients; physically inactive patients; and those with left ventricular hypertrophy, diabetes mellitus, chronic renal disease, metabolic syndrome, and high sodium intake [4].

### Hypertension and inflammatory markers

The long-term effect of RH on the vascular system can be catastrophic. Currently, vascular abnormalities are known to exist early in the development of hypertensive diseases and may manifest as severe endothelial dysfunction.

Endothelial dysfunction is considered an early marker of vascular complications. It participates in the pathophysiology of atherogenesis, which occurs in one of the first stages of arterial disease [6, 7] as a result of risk factors that promote oxidative stress and nitric oxide (NO) inactivation [8]. Endothelial dysfunction involves several functional changes: impaired endothelium-dependent vasodilation, changes in the endothelial barrier function, inflammatory activation, and stimulation of coagulation [9]. Proinflammatory changes seem to be involved in the development of hypertension and in many other situations, including aging, diabetes, dyslipidemia, smoking, and obesity [10–13]. These changes are characterized by a series of highly specific and essentially inflammatory, cellular, and molecular responses that lead to endothelial injury. Environmental factors such as salt intake and psychosocial stress may contribute to the activation of systems that participate in the pathogenesis and progression of hypertension. Moreover, both the sympathetic nervous and renin-angiotensin-aldosterone systems have very important roles, as there are correlations between the activation of these systems and increased inflammatory activity in hypertensive subjects. Thus, hypertension seems to be associated to inflammatory disorders [13]. The inflammatory process is characterized by infiltration of macrophages and T lymphocytes in the endothelium. Activated lymphocytes and macrophages release a variety of inflammatory mediators such as cytokines, adhesion molecules, and matrix metalloproteinases (MMPs), resulting in increased inflammatory cell recruitment, the migration and proliferation of endothelial cells, platelet aggregation, and the release of free radicals [11, 14].

Several inflammatory markers, such as proinflammatory cytokines, interleukins (IL-1β, IL-6, IL-8, IL-18), and tumor necrosis factor (TNF-α) related to activated monocytes, have been identified in these situations. Elevated expressions of intercellular adhesion molecule-1 (ICAM-1) in endothelial cells [11, 15, 16] and highly sensitive C-reactive protein (hsCRP) have also been found. Recent studies show that cardiovascular risk is twice as high in patients with elevated hsCRP compared to patients with lower values. The hsCRP also has an effect on the endothelial surface as the migration and adhesion of monocytes increases, causing the synthesis of chemotactic factors due to the secretion of other proinflammatory factors, such as TNF-α, IL-6, and IL-8 [13, 17]. In addition to proinflammatory cytokines, other cytokines with anti-inflammatory activity, such as IL-10, are present.

In animal studies, IL-10 has demonstrated protective roles against the stability and formation of atherosclerotic lesions. Some clinical studies have shown that patients with acute coronary syndrome have reduced IL-10 levels compared to stable patients, raising the possibility that low levels of IL-10 may be related to atherosclerotic plaque instability [18].

Vascular endothelial dysfunction also promotes inflammation by inducing the production of vasoconstrictor agents, adhesion molecules, and growth factors, including angiotensin II (Ang II). Ang II, one of the end products of the renin-angiotensin system (RAS), is actively involved in the pathogenesis/pathophysiology of hypertension. It may be responsible for triggering endothelial dysfunction and vascular inflammation by inducing oxidative stress, a fact that results in the release of inflammatory mediators and in cell growth [12, 13, 18, 19]. Some studies have demonstrated that Ang II increases the expressions of IL-6, IL-8, IL-18, and the monocyte chemoattractant protein (MCP-1), the last of which is the main regulator of leukocyte recruitment to the vascular wall. In addition, Ang II stimulates the expression of ICAM-1 and the infiltration of macrophages independently of the increase in BP [19, 20].

The role of Ang II as a proinflammatory mediator of vascular injury is supported by the protective anti-inflammatory effects of RAS inhibitors. Among other effects, RAS inhibitors have properties that result in antithrombotic and fibrinolytic actions. The thrombogenic status mainly results from a high concentration of plasminogen activator inhibitor (PAI-1), which leads to an excessive accumulation of fibrin inside vessels, a situation that contributes to vascular events [14]. Thus, PAI-1 is considered an important regulator of thrombogenesis; increased thrombogenesis can increase the risk of atherothrombotic events and promotes the progression of vascular disease [21].

Fibrinogen, an acute phase protein, and cytokines are also directly related to vascular disease [22]. Prospective studies on healthy subjects demonstrated a direct and independent association between the plasma fibrinogen levels and the risk of coronary and cardiovascular events and mortality [16].

As previously mentioned, vascular inflammation and oxidative stress play a crucial role in the pathogenesis of vascular injury mediated by Ang II. Recently, angiotensin-converting enzyme 2 (ACE-2) was identified as having a pleiotropic effect on Ang II, resulting in the neutralization of its proinflammatory and pro-oxidant actions via receptors. Interestingly, the Ang II type-1 receptor blockers may increase ACE-2 expression and activity, thereby reducing cardiovascular and oxidative damage [23].

MMPs are responsible for the degradation of extracellular matrix proteins, breaking them down into their specific peptide bonds, including the cells of the vascular smooth muscle, endothelium, fibroblasts, and inflammatory cells [24–26]. The interaction of MMPs with their endogenous tissue inhibitors (MMP tissue inhibitors - TIMPs) is important among the factors that regulate MMP activity [27–29]. Under physiological conditions, a balance exists in the ratio of MMPs and TIMPs. However, during pathological processes such as hypertension, an imbalance occurs that leads to excessive degradation of extracellular matrix proteins [28] and, consequently, to pathological vascular remodeling. Among these proteolytic enzymes, MMP-9 has an important role in cardiovascular diseases, including atherosclerosis, coronary artery disease, vascular aneurysms, and hypertension [30–36]. Studies suggest that increases in MMP-9 activity are associated with increased arterial stiffness not only in patients with isolated systolic hypertension but also in young individuals without comorbidities [34, 36]. Notwithstanding these reports, studies are scarce that compare MMP levels with arterial stiffness markers in populations with different BP levels.

The aforementioned biochemical changes have a direct relationship with high BP, endothelial dysfunction, and increased vascular stiffness in RH patients, as is demonstrated by reduced flow-mediated vasodilation and increased pulse wave velocity (PWV), which result in higher central BP levels (CBP) [37].

### Central hemodynamics, sympathetic inhibition and hypertension

The CBP has a predictive value in the risk stratification of cardiovascular morbidity and mortality as demonstrated by the CAFE (Conduit Artery Function Evaluation) study. This study showed different effects on the CBP of groups treated with amlodipine and atenolol even though reductions in peripheral arterial pressure were similar. This difference has been suggested to be responsible for the favorable cardiovascular outcomes in the amlodipine group of the ASCOT study (Anglo-Scandinavian Cardiac Outcomes Trial), which demonstrated the importance of CBP control in relation to peripheral BP and how it can be influenced by certain drug therapies [38].

The CBP is directly influenced by arterial stiffness; the arteries, especially the aorta and carotid, are estimated to stiffen by about 10 to 15 % in men and 5 to 10 % in women each decade [39]. Arterial stiffness is a major determinant for the increase in the pulse pressure (PP) and CBP, variables considered risk predictors of myocardial infarction, stroke, and heart failure. In addition, higher cardiovascular morbidity and mortality have been associated with increased CBP, especially in hypertensive diabetics, the elderly, and chronic renal disease patients [40, 41]. Thus, advanced age and high BP are the two most important variables for increased arterial thickness and, consequently, arterial stiffness.

The influence of arterial stiffness on the CBP explains why the pulse wave, generated in every left ventricular ejection period, propagates cyclically throughout the arterial tree to the peripheral arteries. Changes in the impedance and structural or geometric discontinuity of the arterial tree along its path generate reflected waves that move backwards into the ascending aorta and to the heart (backward wave). Therefore, the CBP is the sum of the anterograde and retrograde (reflected wave) components [42, 43]. Wave propagation is amplified from the central aorta to the peripheral arteries. That is, the amplitude of the pressure wave will be greater in peripheral arteries than in the central arteries, which explains the increased pressure in the brachial artery compared to the aorta (central) in younger individuals (the CBP is about 10 to 20 mmHg lower than the peripheral BP). With aging, a loss occurs in arterial elasticity, increasing both the arterial stiffness and the PWV. Increased PWV causes a faster return of the reflected pulse wave with early overlap of the reflected wave during systole (unlike what happens in healthy young people when the reflected wave returns later in systole). This leads to an increase in the systolic pressure and elevation in central PP (PP amplification), which, in turn, increases the afterload on the left ventricle and reflects an increase in the CBP, which almost equals the peripheral BP [43, 44]. This increase in the CBP caused by the reflection of waves is known as the augmentation index (AIx). Hence, the AIx is an alternative index derived from the analysis of the central aortic pressure curve and quantifies the effect of reflected waves [45–47]. It has the advantage of taking into account the time of anterograde and retrograde waves, which are the main determinants of CBP.

Currently, nonpharmacological therapeutic methods are being used to treat RH. These are invasive, require long-term follow up to monitor the results and may cause unwanted complications during and after the procedure, besides being expensive. Examples of these methods are denervation or ablation of the renal sympathetic ganglia and chronic carotid baroreflex stimulation [48]. Both these techniques have generated good results in the reduction of peripheral BP by improving vasomotor tone related to sympathetic suppression; however, similar reductions in BP were reported for treatment and placebo groups [48, 49]. Thus, the identification of new noninvasive therapeutic approaches to control RH is essential. Transcutaneous electrical nerve stimulation (TENS) may be a new, easy to apply, reproducible, and inexpensive technique for this purpose. TENS is capable of inhibiting primary afferent pathways using low-frequency electrical pulses generated by electrodes attached to the skin, which cause local numbness and thus inhibit pain [50, 51]. In addition to the analgesic effects, electrical stimulation has been shown to enhance a local vasodilator effect, which may contribute to reducing BP and prevent ischemia [52]. Among the mechanisms that might explain this anti-ischemic action are the inhibition of sympathetic segmental vasoconstriction, release of vasodilatory peptides from sensory neurons, and the pump effect of muscle contractions [53–56]. Other favorable effects have been observed on applying TENS in the cervicothoracic ganglion region. The cervicothoracic ganglion or stellate ganglion, whose predominant action is sympathetic activity, is a confluence of nerves located in the posterior cervical region at the junction of the lower and upper thoracic cervical ganglia [53–58].

Mannheimer et al. demonstrated that low-frequency electrical stimulation applied to the suprathoracic lymph node region produced an anti-ischemic effect by reducing myocardial oxygen demand due to the reduction in the afterload resulting from a reduction in BP [53]. This response, which is hypothetically based on the observed reduction in epinephrine and norepinephrine levels, may involve a reduction in sympathetic nervous system activation [53, 57]. In turn, Silva et al. observed that TENS applied to the suprathoracic ganglion region during exercise might be associated with decreased peripheral and central BP in healthy young people, a response indicated by significant changes in heart rate variability [58].

Given the scarcity of publications in the scientific literature, this innovative work will study the effects of the application of TENS in the suprathoracic lymph node region to reduce peripheral BP and CBP in RH patients.

### Rationale

Due to the high incidence of cardiovascular morbidity and mortality caused by RH, the introduction of new, more effective, highly specific, and noninvasive therapeutic approaches that are reproducible and less costly from a financial point of view are needed.

### Research question

The following research questions will be explored:

- Does TENS constitute a new therapeutic option in the reduction of peripheral blood pressure of patients with resistant hypertension?
- Does TENS constitute a new therapeutic option in the reduction of central pressure of resistant hypertensive patients?
- Does TENS reduce arterial stiffness measurement parameters?
- Can TENS positively influence metabolic disorders that involve the progression of arterial stiffness?

### Objectives

The research objectives of this study are as follows:

1. To evaluate the effectiveness of TENS as antihypertensive therapy in RH patients
2. To assess the peripheral and central BP before and after the use of TENS
3. To evaluate the behavior of arterial stiffness by measuring the AIx and PWV before and after the use of TENS
4. To observe the biochemical changes, inflammatory markers, and the participation of RAS inhibitors before and after the application of TENS

## Methods/design

### Design

The present trial (clinicaltrials.gov identifier: NCT02365974, registered on 8 February 2015) will be a randomized, open-label, parallel-assignment study controlled with a sham group. All patients who participate in the research will sign informed consent forms. The project design, approved by the Research Ethics Committee of FAMERP, São Paulo, Brazil, which is accredited by the Office of Human Research Protections as an Institutional Review Board (CAAE n° 07606212.5.0000.5415, n° 94.248/2012) (Additional file 1), will comply with the following described criteria.

### Inclusion criteria

The inclusion criteria are as follows:

- RH patients taking at least three antihypertensive drugs at full doses, one of which is preferably a thiazide diuretic
- Age between 40 and 70 years

### Exclusion criteria

The exclusion criteria are as follows:

- Use of a cardiac pacemaker
- Cardiac electrical conduction abnormalities evidenced by ECG or stress test (ergometry)
- Dermatological abnormalities at the site of the application of TENS
- Uncontrolled diabetes in patients using oral hypoglycemic agents or insulin with glycated Hb ≥ 7.5 %
- Secondary hypertension
- Patients who do not adhere to the lifestyle changes recommended by their physician such as low-sodium diet and weight loss
- Moderate to severe cervical-thoracic scoliosis
- Obesity with body mass index (BMI) ≥ 35 kg/m^2^

### Random allocation

A computer-validated software program (Random Allocation) will be used for the random allocation. The study coordinator will organize and number the envelopes, which will be allocated in order of patient enrollment. The professional responsible for the Endo-PAT procedure and evaluation of arterial stiffness parameters will be blinded.

### Interventions

**Fig. 1:**
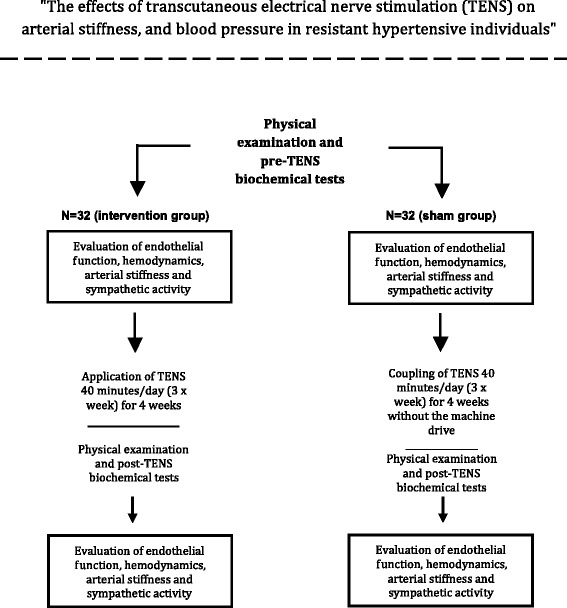
Flowchart of study

TENS will be applied in the cervicothoracic ganglion region in the active comparator group, and the sham group will not be submitted to transcutaneous electrical stimulation. Figure 1 shows a flowchart of the selection of participants and interventions.

### Randomization and follow‐up

#### Protocol

Patients will be divided into two groups: treatment and control (sham group). The sham group will be submitted to the procedure to fit the stimulation equipment. However, these patients will not be submitted to stimulation. Both groups will undergo a physical examination at the start and end of the study period to determine anthropometric variables. The BMI, defined as the weight (kg) divided by the patient’s height squared (m^2^), will be calculated. The height will be determined in centimeters using a measuring tape while the individual stands in bare feet. Individuals with a BMI < 25 kg/m^2^ will be classified as normal, overweight as a BMI between 25 and 30 kg/m^2^, and obese as a BMI ≥ 30 kg/m^2^. The waist circumference will be measured using a measuring tape at half the distance between the iliac crest and the lower costal margin. Patients will undergo the following tests before and after the end of the 4-week study period: 24-h ambulatory blood pressure monitoring (ABPM), 24-h dynamic electrocardiography, continuous measurement of beat-to-beat BP, endothelial function measurement (Endo-PAT 2000), and radial artery applanation tonometry (AT). Table 1 shows a summary of the key practical aspects of the study with all follow-up visits and requested exams, and Fig. 1 represents the flowchart of the study.

**Table 1:** Key practical aspects of the study with all the clinical visits and the requested exams

Visits	Screening	1^st^ week	2^nd^ week	3^rd^ week	4^th^ week
Weeks	−1	1	2	3	4
Informed consent	X				
Inclusion and exclusion criteria	X	X			
Medical history	X				
Medical evaluation/physical examination (BP measure)	X	X	X	X	X
Randomization		X			
Application of TENS (3x/week)		X	X	X	X
Creatinine	X				X
Fasting glucose	X				X
Glycated hemoglobin	X				X
Potassium	X				X
Uric acid	X				X
Insulin	X				X
Total cholesterol	X				X
HDL-c	X				X
Triglycerides	X				X
Urinary sodium	X				X
Urinary potassium	X				X
Microalbuminuria	X				X
GFR estimation	X				X
Specific biochemistry tests		X			X
Ambulatory blood pressure monitoring		X			X
24-h electrocardiographic monitoring		X			X
Evaluation of sympathetic activity		X			X
Evaluation of endothelial function		X			X
Evaluation of arterial stiffness		X			X

*BP* Blood pressure, *TENS* transcutaneous electrical nerve stimulation, *HDL-c* high-density lipoprotein cholesterol, *GFR* glomerular filtration rate

### Ambulatory blood pressure monitoring

ABPM will be performed using the Mobil-O-Graph monitoring device (I.E.M. Gmb, Stolberg, Germany) [59]. Monitoring requires patients to maintain their normal daily activities with the BP being measured automatically at 30-minute intervals for an entire 24-h period according to the technical norms of the 5^th^ Guidelines on Ambulatory Blood Pressure Monitoring [60]. The following parameters will be measured and evaluated over 24 h: systolic (SBP) and diastolic BP (DBP), PP, central systolic and diastolic aortic pressure, central PP, AIx, PWV, cardiac output, and peripheral vascular resistance.

The SBP and DBP will be obtained by ABPM with the mean values for the 24-h period, daytime, and nighttime being considered for analysis. RH patients with mean BP values of ≥ 130 × 80 mmHg over 24 h, ≥ 135 × 85 mmHg during wakefulness, and ≥ 120 × 70 mmHg when asleep will be considered. PP will be calculated during the periods (24 h, daytime, and night) using the formula PP = SBP - DBP. The normal nocturnal dip will be defined as a drop >10 % in SBP from wakefulness to the period of sleeping.

### 24-hour electrocardiographic monitoring

The assessment of the effects of the inhibition of sympathetic nerve activity on the cardiovascular system will be by 24-h dynamic electrocardiography (Holter System - Cardios-Light/Cardios) in accordance with the Guidelines for the Evaluation and Treatment of Patients with Cardiac Arrhythmias [61]. The Holter System will be attached to the patient early in the morning to perform a continuous recording of ambulatory electrocardiographic signals over 24 h. The electrode attachment sites will be shaved and cleaned to reduce skin impedance. During the monitoring period, the patients should continue their normal daily activities. All data will be recorded on a digital recorder, with subsequent analysis being carried out by trained professionals. Recordings will be made using three bipolar precordial leads; the CM5 lead is very sensitive in the diagnosis of changes in heart rhythm and for detecting myocardial ischemia [61, 62].

### Evaluation of sympathetic activity (continuous measurement of beat-to-beat blood pressure)

The continuous measurement system of beat-to-beat BP (NEW Finapres® - Finapres Medical System/Medical Biolink) will be used to evaluate the sympathetic activity. Patients will be placed in a quiet room with controlled temperature (22 °C), without having ingested any kind of natural or drug stimulant and without pain or a full bladder [63, 64]. Before the examination, the patient must remain seated and at rest for 15 min. After placement of the cuffs, the arms will be fully supported at the xiphoid process, and the BP will be recorded for 5 min.

### Evaluation of endothelial function

The peripheral endothelial function will be assessed noninvasively by measuring the reactive hyperemia of the vessel using the Endo-PAT system (peripheral arterial tonometry - Endo-PAT 2000; Itamar Medical, Caesarea, Israel) [65]. Patients will be instructed to appear for this exam after 12-h overnight fasting, including from any kind of medicine. The temperature of the examination room will be maintained between 21 °C and 24 °C, and tight clothing, watches, bracelets, and rings that might interfere with blood flow to the arms will be removed. During at least 15 min, the patients will remain sitting or lying in the examination room to ensure a relaxed cardiovascular status [65–67].

A pressure cuff is placed on one arm (study arm), whereas the other serves as a control (control arm). The device, comprising a pneumatic plethysmograph, is placed on the tip of each index finger, and a uniform pressure is applied to the distal digital surface allowing the measurement of changes in the pulse volume. The insufflation pressure of the digital device is electronically set at 10 mmHg below the DAP or at 70 mmHg. After a 10-min regulation period, the BP cuff is inflated to 60 mmHg above systolic pressure or to 200 mmHg for 5 min. The device continues to record for 10 min after the cuff is deflated. The absence of residual pulsatility is monitored in the occlusion period. The hyperemia after the period of occlusion stimulates endothelium-dependent vasodilation, causing an increase in the amplitude of the digital pulse. Changes in the pressure signals are filtered, amplified, recorded electronically, and analyzed using an automated computerized algorithm (Itamar Medical). The change from the baseline measurement is defined as the reactive hyperemia index (RHI), which will reflect, in part, the vasodilator function of the digital microcirculation. Endothelial dysfunction is defined as a mean value of 2.34 ± 0.33 less two standard deviations of 20 asymptomatic, healthy control subjects with no history of cardiovascular disease and without major risk factors (RHI ≤ 1.68) [67].

### Evaluation of arterial stiffness

The arterial stiffness will also be evaluated by AT of the radial artery, as suggested by taking an average of three measurements after the pressure is stabilized. The HEM9000 AI device (OMRON Healthcare, Co. Kyoto, Japan) will be used for this exam.

The AT examination will be performed in a quiet controlled environment (temperature between 21 °C and 24 °C) after the patient has rested for at least 5 min while sitting with the legs uncrossed, the bladder empty, and away from acute stressors. The patient will not have smoked at all for at least 30 min or ingested alcohol. The OMROM AT equipment uses a radial ultrasonic transducer and cuffs with the correct size for the arm circumference, as recommended by the guidelines to evaluate BP [1].

### Application of TENS

TENS will be applied in the cervicothoracic ganglion region located between the C7 and T4 vertebral processes for 40 min three times weekly for a total of 4 weeks.

**Fig. 2:**
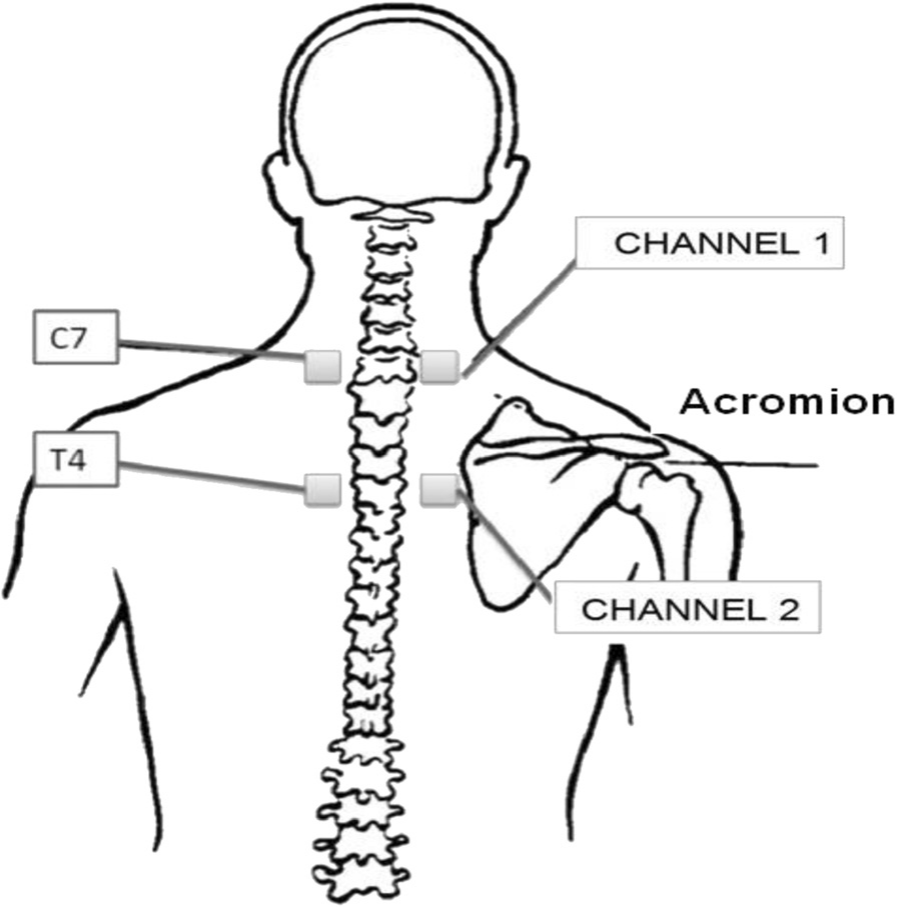
Diagram showing the location for application of the transcutaneous electrical nerve stimulation (TENS)

Electrostimulators (Endomed 684, Enraf Noniuns, Rotterdam, Netherlands) with two 80-Hz output channels at a pulse width of 150 μs will be used for the application of TENS. The intensity in milliamps (mA) will be adjusted depending on the sensitivity of each individual patient. This electrical stimulus has a high-frequency current that will cause mild local paresthesia without causing pain or unpleasant sensations. The intensity will be increased from zero until the perceived sensation reaches the maximum sensory threshold without pain or discomfort or involuntary muscle contractions. The 5 × 5-cm self-adhesive electrodes (Valutrode mark) are attached to the patient after marking the correct location using a dermatographic pencil and cleaning the skin. The arrangement thereof will be parallel on each side of the C7 (channel 1) and T4 (channel 2) vertebral spinous processes (Fig. 2) [58].

### General biochemistry assessment

Biochemical tests will be performed at two time points, before and after the 4-week study period, and include blood sugar, glycated hemoglobin, total cholesterol (TC), high-density lipoprotein cholesterol (HDL-C), triglycerides (TG), creatinine, potassium, uric acid, and plasma insulin levels.

Blood sugar, TC, HDL-C, and TG levels will be determined by colorimetry after fasting for 12 h using a RXL Dimension device with the Dade Behring reagent. Diagnosis of diabetes mellitus (DM) will be established by patient history, the use of hypoglycemic drugs, and measurement of the serum glucose [68]. The LDL-C fraction will be calculated using the formula LDL-C = TC - HDL-C - TG/5 (for TG < 400 mg/dL) [69]. Additionally, the electrochemiluminescence method will be used to measure plasma insulin (immunoassay for the in vitro quantitative determination of human insulin in serum and plasma using the Elecsys 1010/2010 immunoassay and E170 modular analytics analyzers by Roche). The homeostasis model assessment of insulin resistance (HOMA-IR) index will be applied after obtaining blood sugar and insulin levels in order to determine insulin resistance and the functional capability of the pancreatic beta cells. Insulin resistance will be characterized when this ratio exceeds 2.71 [70]. The HOMA-IR index is calculated using the following formula:

$$ \mathrm{HOMA}\hbox{-} \mathrm{I}\mathrm{R}:\;\frac{\mathrm{Fasting}\;\mathrm{insulin}\left(\mathrm{M}\mathrm{c}\mathrm{U}/\mathrm{mL}\right)\times \mathrm{fasting}\;\mathrm{glucose}\left(\mathrm{mmol}/\mathrm{L}*\right)}{22.5}. $$

In order to convert the glucose concentration to mmol/L it is necessary to multiply the value in mg/dL by 0.0555.

Measurement of serum creatinine will be performed by a standardized enzyme-kinetic biochemical test and will be considered high when serum creatinine is > 1.4 mg/dL [71, 72].

Urinary sodium and potassium levels (mEq/L) will be evaluated by flame photometry. Urinary sodium and potassium levels excretion will be calculated by multiplying the urinary sodium and potassium concentrations in mEq/L by the 12-h urine volume [73]. Microalbuminuria will also be evaluated. To evaluate microalbuminuria, the urinary albumin-to-creatinine ratio (UACR) will be obtained from urine samples collected in the morning. Urine creatinine will be calculated using a colorimetric method, and albuminuria will be determined using the nephelometric method. The glomerular filtration ratio (GFR) will be estimated using the modification of diet in renal disease (MDRD) formula: GFR_MDRD_ (mL/min/1.73 m^2^) = 186 (serum creatinine)^−1.154^ × (age)^−0.203^ × (0.742 if female) × (1.212 if black) [74].

### Specific biochemistry tests

Specific biochemical tests will be considered (Table 2): serum aldosterone (AS); plasma renin activity (PRA); highly sensitive C-reactive protein (hsCRP); metalloproteinase 9 (MMP-9); tissue inhibitors of metalloproteinase (TIMP-1 and TIMP-2); nitrites and nitrates; interleukin-1, 6, 8, 10, and 18 (IL-1, IL-6, IL-8, IL-10 and IL-18); TNF-α, thromboxane B_2_ (TXB_2_); fibrinogen; intercellular adhesion molecule-1 (ICAM-1); plasminogen activator inhibitor (PAI-1); angiotensin converting enzyme (ACE); and angiotensin II (Ang II).

**Table 2:** Specific biochemistry tests

Inflammatory markers and endothelial dysfunction	
• IL-1, IL-6, IL-8, IL-18 • TNF-α • hsCRP and fibrinogen • intercellular adhesion molecule (ICAM-1)	
Anti-inflammatory	
• IL-10	
Coagulation	
• plasminogen activator inhibitor (PAI-1) • fibrinogen	
Renin Angiotensin Aldosterone System	
• Ang II • angiotensin converting enzyme (ACE) • plasma renin activity • serum aldosterone	
Extracellular matrix and tissue inhibitors	
• MMP-9 • TIMPs	

*IL* interleukin, *Ang II* Angiotensin II, *TNF-α* tumor necrosis factor alpha, *hsCRP* highly sensitive C-reactive protein, *MMP* metalloproteinase, *TIMPs* tissue inhibitors of metalloproteinase

To evaluate the hsCRP, MMP-9, TNF-α, AS, PRA, TIMPs-1 and −2, nitrites and nitrates, IL-1, IL-6, IL-8, IL-10, IL-18, thromboxane B2, and fibrinogen levels, plasma samples will be obtained after centrifugation at 3500 rpm and 4 °C for 15 min immediately after collection and then stored at −80 °C for later analysis. The IL-1, IL-6, IL-8, IL-10, IL-18, TNF-α, MMP-2 and −9, ACE, and Ang II will be measured using the ELISA technique (R & D systems, Minneapolis, MN, USA) according to the manufacturer’s instructions and as described in a previous study [75]. The hsCRP is determined by a highly sensitive assay with a BN II nephelometer (Dade Behring, Marburg, Germany). The multiplex method will be used to measure the PAI-1 and ICAM-1 inflammatory mediators [14]. Plasma concentrations of tissue inhibitors (TIMP-1 and −2) will be measured using a commercially available enzyme-linked immunosorbent assay (R & D Systems, Minneapolis, MN, USA) according to the manufacturer’s instructions.

The routine PRA assay available today is based on the determination of plasma angiotensin I generated in vitro by the proteolytic action of renin on its substrate: angiotensinogen under predetermined conditions of time and temperature. Blood samples should be collected and maintained with special care: (1) in iced syringes and tubes, (2) using EDTA as a proteolysis inhibitor (angiotensinase), (3) separating the plasma in a refrigerated centrifuge no more than 30 min after each collection, and (4) separating and storing the plasma at −20 °C or less (ideally at −70 °C) until the time of the assay. As the PRA levels fluctuate in response to posture, diet, and hydration (in parallel with the aldosterone system), reference values exist for the various conditions, but most laboratories require that the patient has fasted and rested lying down for at least 30 min before the collection of baseline samples [76].

A commercial kit (Cayman Chemical Co. Ann Arbor, MI, USA) will be used under controlled conditions to measure the total nitrite and nitrate concentrations in the biological fluids and by-products of the production of NO using the NO synthase enzyme. Nitrites and nitrates are stable end products of the NO reaction with molecular oxygen. The proportions of nitrates and nitrites are variable and cannot be accurately determined. Therefore, the best indicator of NO production is the sum of the nitrites and nitrates. The Cayman Chemical kit used to determine nitrites and nitrates provides an accurate and convenient method for measuring the total concentrations through a simple two-step process. This test is based on the determination of nitrite, not of nitrate, using the Griess reaction. The method is very simple, and a large number of samples can be processed in a short time, making it quite suitable for routine application in research laboratories. Plasma samples, collected in an EDTA anticoagulant, are first diluted four times using distilled water and de-proteinized by adding 1/20 volume of zinc sulfate (300 g/L), giving a concentration of 15 g/L. After centrifugation at 10,000 g for 5 min or 1000 g for 15 min at ambient temperature, 100 μL of Griess reagent (1 g/L sulfanilamide, 25 g/L of phosphoric acid, and 0.1/L naphthylethylenediamine dihydrochloride) is added. After 10 min incubation at room temperature, the sample develops a violet color, the intensity of which is measured in a plate reader using a 540-nm wavelength [77]. Each sample will be tested in duplicate. The determination of the standard curve of nitrite concentration will be carried out using the protocol provided by the manufacturer. Subsequently, a graph is drawn to map the intensity of readings at 540 nm to known concentrations of nitrates or nitrites. The total nitrate concentrations (nitrites + nitrates), or nitrates and nitrites in isolation, are calculated by determining the intersection of the spectrophotometer reading. The concentration of the sample is subsequently calculated by the simple rule of three.

Thromboxane B2 will be evaluated in blood samples collected in test tubes containing EDTA. The plasma is separated by centrifugation and stored at −20 °C until testing. The plasma samples are purified using a reversed-phase column (Sep-Pak C18 - Waters Co., Milford, MA, USA), and the TXB_2_ plasma levels (metabolite of TXA_2_) are determined using a commercial enzyme immunoassay kit (ELISA Cayman Chemical Co. Ann Arbor, MI, USA). This assay uses 500 μL of venous blood plasma and is based on the competition between TXB_2_ and a TXB_2_ tracer (TXB_2_ attached to an acetylcholinesterase molecule) with a limited number of TXB_2_-specific rabbit antiserum binding sites. The concentration of the tracer is held constant, while the free TXB_2_ changes. The TXB_2_ antiserum complex (free tracer) binds to a rabbit monoclonal antibody previously attached to the plate, which is rinsed to remove unwanted reagents, and Ellman’s reagent is added (which contains the substrate for acetylcholinesterase). The product of this enzymatic reaction has a yellow color and absorbs 420-nm wavelength light in a spectrophotometer [78, 79].

### Primary outcome measure

The primary outcome measure will be the change from baseline in the peripheral blood pressure of individuals with resistant hypertension after transcutaneous electrical nervous stimulation.

### Secondary outcome measures

The secondary outcome measures from baseline are the arterial stiffness measurement parameters (including central blood pressure) of individuals with resistant hypertension after transcutaneous electrical nervous stimulation.

### Assessment of outcomes

Blood pressure (mean of two measurements by an automatic electronic device Omron HEM-711 DLX) and hemodynamic parameters (by Omron HEM 9000 AI device) will be measured in the office at the follow-up visits.

### Adverse events

The adverse events will be investigated by open questions and by a semistructured questionnaire, which will address general symptoms and the presumed adverse effects of the electrostimulation used in the trial. Laboratory adverse events, such as metabolic changes and inflammatory markers, will be investigated at the final visit of the participants.

### Missing or dropout

Participants will be registered with a phone number and address for further contact in case of missing outlined visits.

### Sample size calculation

The site http://www.lee.dante.br/pesquisa/amostragem/amostra.html was used to estimate the sample size. The sample size calculation with a P alpha of 0.05, statistical power of 80 %, standard deviation of 8 mmHg, and maximum acceptable absolute difference of 6 mmHg (diastolic BP), indicated the necessity to study 28 patients per group (intervention versus sham). However, considering a potential 10–15 % dropout or loss to follow-up rate, 32 will be considered for each group. The difference of 5 mmHg (diastolic) has been achieved, on average, in clinical trials that have demonstrated the advantage of a drug over placebo or other nonpharmacological treatment in the prevention of major cardiovascular outcomes.

### Statistics

All analyses will be performed using SPSS Statistical Software (IBM SPSS Statistics for Windows, Version 21.0. Armonk, NY, USA). Continuous variables will be presented as mean ± SD, and categorical variables, as frequencies. Differences between the groups at baseline will be evaluated by an unpaired *t* test or the Mann–Whitney test for comparison of continuous variables. The Chi-square test or Fisher’s exact test will be employed to compare categorical variables. The change from baseline to 4-week follow-up in the groups will be evaluated using the paired *t* test for continuous variables. Pearson’s correlation will be used to assess the relation between BP reduction and treatment (intervention). For all analyses, a two-sided *P* < 0.05 will be considered statistically significant.

## Discussion

Over the decades, RH has become very common and costly. Adequate control requires several drugs, and in many cases, treatment is not successful. This is due to target organ damage or even an autonomic imbalance. Studies have shown that sympathetic nervous system inhibition by both renal denervation and by central inhibition have significant effects in reducing BP; however, these treatments are costly and invasive. Moreover, other studies have shown that sympathetic nervous system inhibition can also be noninvasively achieved by electric current. Even so, the effects of electrical stimulation on the metabolic system still need to be elucidated. Additional file 2 shows the checklist of study protocol data and related documents in accordance with SPIRIT (Standard Protocol Items: Recommendations for Interventional Trials). The final report, and its extension to nonpharmacological interventions, will follow the main CONSORT 2010 guideline.

### Trial status

The randomization of patients has begun.

## Additional files

Additional file 1: Research Ethics Committee. Declaration of Research Ethics Committee of the Medical School in São José do Rio Preto (FAMERP). (PDF 182 kb) Additional file 2: SPIRIT. Checklist of study protocol data and related documents according to SPIRIT (Standard Protocol Items: Recommendations for Interventional Trials). (PDF 105 kb)

## Abbreviations

BP: blood pressure
CVD: cardiovascular disease
AT: applanation tonometry
AIx: augmentation index
RH: resistant hypertension
MMPs: matrix metalloproteinases
TNF-α: tumor necrosis factor
ICAM-1: intercellular adhesion molecule-1
hsCRP: highly sensitive C-reactive protein
IL: interleukins
ANG-II: angiotensin II
RAS: renin-angiotensin system
MCP-1: monocyte chemoattractant protein
PAI-1: plasminogen activator inhibitor
ACE: angiotensin converting enzyme
TIMPs: tissue inhibitors of metalloproteinase
CBP: central blood pressure
ASCOT: Anglo-Scandinavian Cardiac Outcomes Trial
PP: pulse pressure
PWV: pulse wave velocity
TENS: transcutaneous electrical nervous stimulation
Hb: hemoglobin
BMI: body mass index
ABPM: ambulatory blood pressure monitoring
SAP: systolic arterial pressure
DAP: diastolic arterial pressure
RHI: reactive hyperemia index
mA: milliamps
TC: total cholesterol
HDLc: high-density lipoprotein cholesterol
TG: triglycerides
PI: plasma insulin
DM: diabetes mellitus
HOMA: homeostasis model assessment of insulin resistance
SA: serum aldosterone
PRA: plasma renin activity
RIA: radioimmunoassay
NO: nitric oxide
TXB_2_: thromboxane B2
