# Supplementary material for: Effects of transcutaneous electrical nerve stimulation (TENS) on arterial stiffness and blood pressure in resistant hypertensive individuals: study protocol for a randomized controlled trial
Source: Trials. 2016 Mar 29;17:168. doi: 10.1186/s13063-016-1302-8 (PMC4812656; doi:10.1186/s13063-016-1302-8)
Supplement: Additional file 1: — Research Ethics Committee. Declaration of Research Ethics Committee of the Medical School in São José do Rio Preto (FAMERP). (PDF 182 kb) [file 13063_2016_1302_MOESM1_ESM.pdf]

FACULDADE DE MEDICINA DE SÃO JOSÉ DO RIO PRETO
Autarquia Estadual - Lei n.º 8899 de 27/09/94

Parecer nº 94.248

COMITÊ DE ÉTICA EM PESQUISA

O projeto de pesquisa **CAAE 07606212.5.0000.5415** sob a responsabilidade de **José Fernando Vilela Martin**, com o título "Efeitos da estimulação Elétrica Nervosa Transcutânea (TENS) na Rigidez Arterial, Pressão Arterial Periférica e Central em Pacientes Hipertensos Resistentes" está de acordo com a resolução do CNS 196/96 e foi **aprovado por esse** CEP.

Lembramos ao senhor(a) pesquisador(a) que, no cumprimento da Resolução 251/97, o Comitê de Ética em Pesquisa em Seres Humanos (CEP) **deverá receber relatórios semestrais sobre o andamento do Estudo**, bem como a qualquer tempo e a critério do pesquisador nos casos de relevância, além do envio dos relatos de eventos adversos, com certeza para conhecimento deste Comitê. **Salientamos ainda, a necessidade de relatório completo ao final do Estudo.**

São José do Rio Preto, 11 de setembro de 2012.

Prof. Dr. Fernando Batigália
Presidente do CEP/FAMERP
